# Infectious keratitis in pediatric population aged less than two years: a tertiary eye institute experience

**DOI:** 10.1186/s12348-024-00414-0

**Published:** 2024-07-15

**Authors:** Mohammad Soleimani, Seyed Mahbod Baharnoori, Sadegh Ghafarian, Mehrnaz Atighehchian, Kasra Cheraqpour, Seyed Ali Tabatabaei, Hamidreza Ghanbari, Mahdi Soleimanzadeh, Faezeh Moghimpour Bijani, Solmaz Almasi, Haniyeh Zeidabadinejad, Ali Davarpanah, Marzieh Sajedi, Bahram Bohrani Sefidan, Samer Habeel, Ahmad Masoumi, Mohammad Hossein Zamani, Ali R. Djalilian

**Affiliations:** 1 Eye Research Center, Farabi Eye Hospital, Tehran University of Medical Sciences, Qazvin Square, Tehran, 1336616351 Iran; 2 Department of Ophthalmology and Visual Sciences, University of Illinois at Chicago, Chicago, IL USA; 3 Internal Medicine Department, Shariati Hospital, School of Medicine, Tehran University of Medical Sciences, Tehran, Iran

**Keywords:** Keratitis, Pediatric, Infantile, Corneal ulcer, Microbiology, Infectious keratitis

## Abstract

**Background:**

Infectious keratitis is a serious ocular condition, which can lead to corneal scarring, vision loss, and even blindness. Pediatric infectious keratitis accounts for about 13% of all cases, although there is a lack of comprehensive data regarding keratitis in less than two years of age population group. This study was aimed to determine predisposing factors, clinical characteristics, microbial profile, and management of infectious keratitis in a population of children aged less than two years.

**Materials and methods:**

A retrospective study was carried out in a tertiary eye institute over a period of 18 years from July 2005 to December 2022. Collected data was analyzed for demographics, predisposing factors, clinical features, and treatment methods.

**Results:**

Fifty-seven cases of keratitis were identified. Age of the patients ranged from 1 to 24 months (Median: 6, interquartile range: 2–10). Thirty cases were male (52.6%). Predisposing factors were identified in 39 cases (68.4%): consisting of prior ocular trauma (*n* = 15), previous intraocular surgery (*n* = 11), ocular surface disease (*n* = 10), nasolacrimal duct obstruction (*n* = 4), prematurity (*n* = 3), developmental delay (*n* = 2), TORCH infection (*n* = 1), and contact lens (*n* = 1). Corneal thinning was observed in 29 eyes (50.9%), which progressed to perforation in 13 eyes (22.8%). Three patients developed endophthalmitis (95% CI, 1.5–13.4%). Most eyes had negative smear (60.4%) and culture (59.6%) results. *Pseudomonas aeruginosa* was the most common microorganism (11 of 21). *Candida albicans* was isolated in one case. In vitro susceptibility results showed good coverage of the combined ceftazidime and vancomycin regimen (100%). Surgical procedures were carried out in 35 eyes (61.4%) and 15 eyes required tectonic procedures (26.3%).

**Conclusion:**

Despite good coverage of medical treatment over cultured isolates, surgical tectonic intervention was required in nearly a quarter of cases to resolve the corneal infection. This finding indicates the necessity of prompt patient referring, corneal sampling and initiation of the treatment.

## Background

Infectious keratitis is a serious ocular condition, which can lead to corneal scarring, vision loss, and even blindness. Pediatric infectious keratitis accounts for about 13% of all cases, although there is a lack of comprehensive data on keratitis in children under two years of age [1]. Despite its rarity, keratitis in infants and toddlers is a significant public health concern, particularly in developing countries.

The most common risk factor for pediatric infectious keratitis is corneal trauma, particularly in developing countries [2–4]. Diagnosis and investigations are more challenging in children, and examination under anesthesia or deep sedation is often required to obtain corneal scrapings and to document the extent of the ulcer.

Despite numerous studies investigating pediatric infectious keratitis, there is a scarcity of literature directly addressing the infantile and toddlers age group in infectious keratitis, with existing papers primarily consisting of case reports [5]. The purpose of this study is to investigate the risk factors, clinical characteristics, treatment modalities, causative microbial pathogens and the antibiotic susceptibility pattern of infectious keratitis among children under two years of age. The study was conducted over an 18-year period at a tertiary eye care center in Iran.

## Materials and methods

Our study was conducted as a retrospective, non-comparative, observational case series. The institutional review board of Tehran University of Medical Sciences confirmed that no ethics approval is required. The study included all cases of infantile and toddlers’ keratitis that were diagnosed and treated at Farabi eye hospital from July 2005 to December 2022. Cases were included in the study if they met the following criteria: age at diagnosis of less than 2 years and evidence of corneal epithelial defect and stromal infiltration on slit-lamp examination. Patients with isolated suspected viral keratitis, according to clinical evidence, were excluded.

All patients were admitted and underwent exam under anesthesia (EUA). The selection of the anesthesia technique (e.g., general anesthesia or minimal sedation) was determined according to the overall health status and initial assessment of the ulcer. If additional surgical measures were to be performed, the induction of general anesthesia was administered. Alternatively, for the sole purpose of corneal scraping, the patient received sedation. Data for each patient was recorded including, age, gender, predisposing factors, associated ocular conditions, and other systemic diseases. Clinical findings such as corneal thinning or perforation, hypopyon, and size of infiltrate, along with microbial results and details of medical and surgical treatments were also recorded. The size of corneal infiltration was measured as the longest diameter in millimeters and was categorized as small if less than 2 mm, medium if between 2 and 6 mm, or large if more than 6 mm.

For laboratory diagnosis, corneal scrapings were obtained from the base and edge of the ulcer using separate sterile surgical blades (#15) under general anesthesia. The obtained samples were used for Gram stains, 10% potassium hydroxide wet mount, as well as culture and antibiogram. Gram‑stained smears were examined at ×400 and ×1000 magnification; the KOH preparations were examined at ×200 and ×400 magnification under a light microscope. For culturing, the material was inoculated in Chocolate agar and Sabouraud dextrose agar (SDA) and was incubated at 37 °C. Cultures were examined daily during the 1st week, twice-weekly for the next 3 weeks. Notably, the cultures were monitored for 3–4 weeks to evaluate any fungal growth. All laboratory methods were performed under standard protocols. Antibiotic susceptibility testing was performed using the disk diffusion method.

The eyes were treated initially based on clinical evaluation and microbiological smear examinations. The initial treatment protocol was instilling topical fortified antibiotic drops on an hourly basis as an empiric treatment. The subsequent treatment strategy was determined based on clinical response and antibiotic susceptibility. In cases of polymicrobial keratitis, treatment was modified to target all identified pathogens. Adjuvant therapy of cycloplegics was given to all patients. Indications for adjunctive surgical procedures such as cyanoacrylate glue application, corneal patch graft, or therapeutic penetrating keratoplasty (PKP) were assessed by an experienced cornea specialist.

Descriptive statistics were done using Excel 2019 (Microsoft Corp.) and data were presented as mean ± SD, median (range) and frequency (%).

## Results

In total, 57 infants with keratitis were identified. Age at diagnosis ranged from 1 to 24 months with a median age of 6 months (interquartile range: 2–10 months). 30 cases were male (52.6%) and 27 were female. The right eye was affected in 22 cases (40.7%), the left eye in 26 cases (48.1%), and bilateral keratitis was observed in 6 cases (11.1%). Laterality data was missing in 3 patients. The patients were hospitalized on an average of 5.33 ± 1.76 days (range: 2–9 days) and after control of the infection they were discharged.

Predisposing factors were identified in 39 cases (68.4%), and 7 patients had two or more risk factors. Risk factors associated with keratitis are summarized in Table 1. History of ocular trauma was the most common risk factor identified in this study, comprising 15 of 57 patients (26.3%). Among them, 9 had unspecified ocular trauma (15.8%), 5 had corneal penetrating injury (8.8%), and one patient had prior thermal burn (1.8%). The unspecified trauma caused by nail in 3 cases, vegetative matter, knife, needle, and falling in one case each, and unknown etiology in two cases. The penetrating injury is also caused by a fork, wood, nail, pliers, and falling in each case. Additionally, 11 patients had previous intraocular surgery (19.3%). The third most common predisposing factor was preexisting ocular surface disease in the affected eye of 10 patients (17.5%), including severe dry eye disease in 5 cases (8.8%), lagophthalmos in 4 cases (7%), and corneal anesthesia in one case (1.8%). Regarding dry eye disease, the etiology was not documented and in the case of corneal anesthesia, work-up for trigeminal hypoplasia was planned; however, the result was unavailable. Other identified risk factors are listed in Table 1.

**Table 1:** Predisposing factors for development of infantile keratitis (*n* = 57)

Risk factor	Number of eyes (*n*)
History of ocular trauma	15 (26.3%)
Unspecified trauma	9
Penetrating injury	5
Thermal burn	1
Previous intraocular surgery	11 (19.3%)
Trabeculectomy	5
Cataract surgery	3
Vitrectomy	3
Ocular surface disease	10 (17.5%)
Severe dry eye	5
Lagophthalmos	4
Corneal anesthesia	1
NLDO	4
Prematurity	3
Developmental delay	2
TORCH	1
Contact lens use	1

All patients had corneal infiltration ranging from 1 to 8 mm in its longest diameter (mean size 3.53 ± 1.51 mm). The size of corneal ulcerations was characterized as small (< 2 mm at its greatest dimension) in 3 eyes, medium (2–6 mm) in 52 eyes, and large (> 6 mm) in 2 eyes. Location of the infiltration on the cornea was central in 26 eyes (45.6%), paracentral in 19 eyes (33.3%), and peripheral in 12 eyes (21.1%). Corneal thinning was observed in 29 (50.9%) eyes, which progressed to perforation in 13 cases (22.8%, 95% CI, 13.4–34.9%). In 22 patients (38.6%), hypopyon was seen at presentation.

During the course of admission, 3 cases (5.3%) developed endophthalmitis (95% CI, 1.5–13.4%). All of these cases had previous intraocular surgery and presented with perforated corneal ulcer and hypopyon at initial presentation. Among patients with a history of previous intraocular surgery, endophthalmitis developed in 27.3% (3 out of 11). One of these cases was a 5-month-old male infant with a history of cataract surgery in his right eye, who presented with perforated corneal ulcer and endophthalmitis two days after surgery. Microbial culture of the cornea showed *Streptococcus pneumoniae*, which ultimately resulted in evisceration due to corneoscleral melting of the eye. The other case involved a 17-month-old male toddler with a history of trabeculectomy in his left eye 66 days before the presentation. In the EUA, which was performed one month after surgery, there was no keratitis, and he was receiving dorzolamide-timolol combination drops and erythromycin ointment, and the corneal microbial culture result showed *Streptococcus pneumoniae*. The last case was a 4-month-old female infant with a history of vitrectomy in her right eye 16 days before the presentation and the culture result was *Candida albicans*.

Corneal scraping smears were negative in 32 eyes (60.4%), while Gram-negative bacilli were observed in 7 eyes (13.2%), Gram-positive diplococci in 7 eyes (13.2%), Gram-positive cocci in 5 eyes (9.4%), and Gram-positive bacilli in 2 eyes (3.8%). No mycelium was observed. Smear results were missing in 4 cases. The in vitro culture results of corneal scrapes showed no growth in 31 eyes (59.6%), *Pseudomonas aeruginosa* was present in 11 eyes (21.2%), *Streptococcus pneumoniae* in 3 eyes (5.8%), *Streptococcus viridans* in 3 eyes (5.8%), *Staphylococcus aureus* in 2 eyes (3.8%), *Staphylococcus epidermidis* in one eye (1.9%), and *Candida albicans* in one eye (1.9%). Culture results were missing in 5 cases. Antibiogram results for each microorganism are reported in Table 2. Among *Pseudomonas aeruginosa* isolates, which were the most common cultured microorganism, all were sensitive to amikacin, gentamicin, ceftazidime, and imipenem. Additionally, 89% were sensitive to levofloxacin, 82% to ciprofloxacin, and none to chloramphenicol, cefazolin, TMP-SMX, or vancomycin. Except for one *Streptococcus pneumoniae* isolate, none of the Gram-positive cocci were resistant to vancomycin. Antibiograms showed that the combined topical regimen of ceftazidime and vancomycin could cover all the cultured isolates, whereas the combined topical cefazolin and amikacin regimen was adequate in 14 of them (70%).

**Table 2:** Surgical procedures (*n* = 57). *One case needed re-PKP in her course of admission

Surgical procedure	Number of eyes (*n*)	Percentage (%)
Tarsorrhaphy	17	29.8
Cyanoacrylate glue	12	21.1
AMT	11	19.3
PKP	8	15.8
Punctal cautery	5	8.8
Corneal patch graft	4	7.0
Keratoprosthesis + vitrectomy	3	5.3
Evisceration	1	1.8

All patients were admitted, and their medical management mainly consisted of topical amikacin (20 mg/ml) in combination with topical cefazolin (50 mg/ml) in 35 eyes (61.4%) until specific antibiogram testing was performed, and the topical drops were modified accordingly. Initial treatment consisted of topical ceftazidime (50 mg/ml) combined with topical vancomycin (50 mg/ml) in other 19 eyes (33.3%). Two eyes were initially treated solely with topical levofloxacin (5 mg/ml) and one eye with antifungal agents. Topical levofloxacin (5 mg/ml) and topical ciprofloxacin (3 mg/ml) were added in 4 and 2 eyes, respectively.

In this series, medical management proved effective in 22 cases (38.6%), while 35 eyes (61.4%) needed one or more surgical procedures in the course of admission (Table 3). Medical treatment alone was sufficient in 54.3% of eyes treated empirically with the combined topical regimen of cefazolin and amikacin (19 from 35 eyes), 10.5% of eyes treated empirically with the combined topical regimen of ceftazidime and vancomycin (2 from 19 eyes), and 50% of eyes treated empirically with levofloxacin (1 from 2 eyes). It should be noted the treatment regimen was change in the course of admission according to antibiogram results. The most common surgical procedure was tarsorrhaphy in 17 eyes (29.8%). Corneal melt and thinning, with or without perforation, required the application of cyanoacrylate glue in 12 eyes (21.1%) and corneal patch graft in 4 eyes (7%). 11 patients (19.3%) with persistent epithelial defect were treated using amniotic membrane transplantation (AMT). Therapeutic penetrating keratoplasty (PK) was performed in 8 eyes (14%), one of them required reoperation due to graft melting. Punctal cauterization was performed on all 5 eyes with severe dry eye (8.8%). 3 eyes with endophthalmitis underwent pars plana vitrectomy through a temporary keratoprosthesis, associated with the subsequent corneal graft (5.3%), and one of these cases ultimately required evisceration.

**Table 3:** Susceptibility results of cultured isolates for common antibiotics

Isolate	Eyes	Susceptibility Result									
		Amikacin	Ceftazidime	Gentamicin	Chloramphenicol	Cefazolin	Ciprofloxacin	TMP-SMX	Vancomycin	Levofloxacin	Imipenem
Pseudomonas aeruginosa	11	100% (11/11)	100% (11/11)	100% (11/11)	0% (0/7)	0% (0/6)	82% (9/11)	0% (0/6)	0% (0/3)	89% (8/9)	100% (7/7)
Streptococcus viridans	3	-	-	67% (2/3)	100% (3/3)	-	100% (1/1)	0% (0/3)	100% (3/3)	100% (3/3)	-
Streptococcus pneumoniae	3	0% (0/2)	50% (1/2)	50% (1/2)	100% (3/3)	100% (2/2)	100% (2/2)	50% (1/2)	67% (2/3)	50% (1/2)	-
Staphylococcus aureus	2	0% (0/2)	0% (0/1)	0% (0/2)	50% (1/2)	0% (0/2)	0% (0/2)	0% (0/2)	100% (2/2)	-	-
Staphylococcus epidermidis	1	100% (1/1)	100% (1/1)	100% (1/1)	100% (1/1)	100% (1/1)	100% (1/1)	0% (0/1)	100% (1/1)	-	-
Overall sensitivity To the antibiotic		75% (12/16)	86.7% (13/15)	78.9% (15/19)	50% (8/16)	27.3% (3/11)	76.5% (13/17)	7.1% (1/14)	66.7% (8/12)	85.7% (12/14)	100% (7/7)

Surgical tectonic procedures, including corneal patch graft, penetrating keratoplasty and keratoprosthesis, were required in 15 cases (26.3%), out of which corneal smear and culture were positive in 8 (53.3%) and 13 (86.7%) cases, respectively. The cultured microorganisms in eyes which needed penetrating keratoplasty were *Pseudomonas aeruginosa* in 5 cases, *Streptococcus viridans* in 2 cases, and no growth was observed in one case.

## Discussion

Infectious keratitis is a rare but devastating ocular disease. Although the prevalence of infectious keratitis is lower in infants compared to adults, it may have a more devastating effect due to the potential for deprivation amblyopia. Additionally, diagnosis and treatment in this age group is challenging due to their inability to communicate and cooperate during examinations, corneal scrapes, and administration of topical medication. Notably, the occurrence of childhood blindness has a negative impact on the development of neurobehavioral functions, consequently compromising the overall quality of life for both children and their families. The significance of addressing childhood blindness lies not only in the existence of preventive measures, but also in the substantial duration of blindness experienced by affected individuals [1]. Previous studies have mostly focused on children under 16 years of age, also including infants among them [3, 4, 6–15]. One study reported kids under 5 years of age [16], while another reported infectious keratitis in the neonatal age group [17]. However, to the best of our knowledge, there is currently no study directly addressing our study age group in the literature.

In this series, ocular trauma was found to be the most common predisposing factor for the development of infectious keratitis, which is consistent with previous studies among the pediatric age group [1, 3, 8–12, 14, 18]. Other common risk factors include previous intraocular surgery and ocular surface problems. Other studies have found contact lens wear to be the most common predisposing factor [19–23], with ocular trauma as the second leading risk factor. Contact lens wear was also the most common in a study performed by Hsiao et al. [23]. However, when categorizing cases into two groups based on age (over or under 12 years), trauma was found to be the more prevalent risk factor in the younger kids. Furthermore, in a study investigating the causes of ocular trauma in infants, self-infliction by the child’s hand was the most common cause of such injuries. Moreover, among these patients with ocular trauma, approximately 25% were found to have infectious keratitis [24, 25].

In our study, approximately half of the eyes had corneal thinning and a quarter had corneal perforations. The high rate of corneal thinning and perforation shows that there is substantial delay in the referral system among local care givers, which will end up in more surgical procedures. Other studies reported the need for surgical intervention in 6%-77.6% of pediatric cases [1, 6, 7, 11, 15, 16, 23]. Surgical tectonic procedures were required in 15 of the cases in our study (26.3%), of which corneal cultures were positive in 86.7%. It appears that when a viable pathogen can be isolated from corneal scrapes, the prognosis tends to be worse. Therapeutic penetrating keratoplasty (TPK) was performed in 8 eyes (14%), and one of them required reoperation due to graft melting. The rate for TPK was reported from zero to 16.9% ^9,14,16,19,23^. Post-keratitis endophthalmitis was observed in 3 eyes that underwent keratoprosthesis placement and vitrectomy with subsequent corneal grafting. The rate of endophthalmitis among eyes with a history of intraocular surgery was 27.3%, and one of those cases ultimately resulted in evisceration.

Approximately 60% of corneal scrapes resulted in an absence of microorganisms. Corneal biopsy or repeat of corneal scraping was not required in none of the cases with negative culture and all of them responded to the treatment, whether medical (29 eyes) or surgical (2 eyes). The culture positivity rates ranged from 34% to as high as 88.8% in cases where the scrapings were obtained under anesthesia [3, 14, 16, 20]. One possible explanation could be the need for general anesthesia to perform the sampling and prior initiation of topical antibiotics. *Pseudomonas Aeruginosa* (21.2%) was the most frequent bacterial agent isolated from corneal scrapes in this series, followed by *Streptococcus spp.* (11.6%). In 2020, Soleimani et al. reported that *S. epidermidis* and *P. aeruginosa* had an equal frequency (10.8%) and were the most frequent bacterial agents [16]. *P. aeruginosa* was found to be the most common pathogen in many studies [1, 8, 9, 12, 23], while others reported *S. epidermidis* as the most common causative agent in pediatric cases [6, 7, 11, 15]. In another study, Al Otaibi et al. reported *S. pneumoniae* as the most common agent [10]. The prevalence of infectious fungal keratitis is 3–37% of all cases in previous studies [7–9, 16, 25, 26], but in our study, only a single case of keratitis induced by *Candida albicans* was observed.

In a study published in 2003, Jeng et al. proved about 75% of corneal ulcers were responsive to monotherapy with fluoroquinolones [27]. Another study reported no significant difference in the outcome between monotherapy with fourth generation fluoroquinolones and combination therapy with fortified antibiotic drops in the treatment of bacterial keratitis [28]. In our study, two eyes received just levofloxacin as the empiric treatment, of which one needed surgical procedure. None of the cases had any corneal thinning or hypopyon. We do not recommend monotherapy in this age group. Among the available cultures, 86.7% of isolates showed sensitivity to aminoglycosides. The sensitivity rates for ciprofloxacin, vancomycin, and chloramphenicol were 76.5%, 66.7%, and 50% respectively. Cefazolin exhibited a high rate of resistance (72.7%). Based on the antibiograms, the combined topical regimen of ceftazidime and vancomycin could effectively cover all the cultured isolates, whereas the combined topical cefazolin and amikacin regimen was adequate in 14 of them (70%).

Our center supports a wide range of distances and occasionally accepts patients from over 1000 km distant. We accept such complicated and challenging patients from all areas of the country. So, we can claim that our reports and statistics are representative of Iran’s condition. One limitation in this series is that our institute is a tertiary eye care center, which may explain the high rate of severe keratitis and the need for surgical procedures, limiting the generalizability of our results. Additionally, the lack of follow-up examinations makes it impossible to comment on prognosis. Also, information pertaining to the time elapsed between beginning of symptoms and initial treatment and referring and also treatment received before the patients’ arrival at our hospital is unavailable.

## Conclusion

In conclusion, keratitis in early stages of life is a serious condition that can lead to significant visual impairment. This study has identified a history of ocular trauma as the most common predisposing factor, followed by previous intraocular surgery and ocular surface problems. Corneal thinning occurred in nearly half of the cases, prompting meticulous ophthalmic examination under general anesthesia as soon as possible.

## Data Availability

Data sets analyzed during the current study are available from the corresponding author upon request.

## Abbreviations

TORCH: Toxoplasmosis, others (syphilis, hepatitis B) rubella, cytomegalovirus, herpes simplex
EUA: Exam under anesthesia
SDA: Sabouraud dextrose agar
PKP: Penetrating keratoplasty
AMT: Amniotic membrane transplantation
TPK: Therapeutic penetrating keratoplasty
